# Boolean Circuits in Colloidal Mixtures of ZnO and Proteinoids

**DOI:** 10.1021/acsomega.4c02468

**Published:** 2024-10-01

**Authors:** Raphael Fortulan, Noushin Raeisi Kheirabadi, Panagiotis Mougkogiannis, Alessandro Chiolerio, Andrew Adamatzky

**Affiliations:** † Unconventional Computing Laboratory, UWE, Bristol BS16 1QY, U.K.; ‡ Bioinspired Soft Robotics Laboratory, Istituto Italiano di Tecnologia, Via Morego 30, Genova 16165, Italy

## Abstract

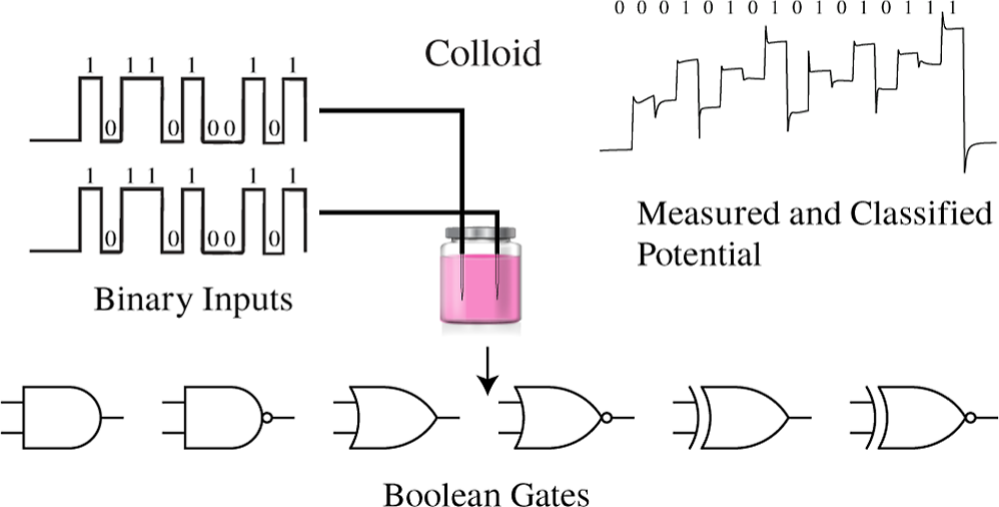

Liquid computers use incompressible fluids for computational processes. Here, we present experimental laboratory prototypes of liquid computers using colloids composed of zinc oxide (ZnO) nanoparticles and microspheres containing thermal proteins (proteinoids). The choice of proteinoids is based on their distinctive neuron-like electrical behavior and their similarity to protocells. In addition, ZnO nanoparticles are chosen for their nontrivial electrical properties. Our research demonstrates the successful extraction of 2-, 4-, and 8-bit logic functions in ZnO proteinoid colloids. Our analysis shows that each material has a distinct set of logic functions and that the complexity of the expressions is directly related to each material present in a mixture. Our study shows that 2-, 4-, and 8-bit logic functions can be successfully extracted from ZnO proteinoid colloids. These findings provide a basis for the development of future hybrid liquid devices capable of general-purpose computing.

## Introduction

Liquid-based computers use incompressible fluids (i.e., substances featuring zero divergence of the flow velocity) as a medium for driving or hosting computational processes. Examples of liquid computers include hydraulic algebraic machines,^1,2^ hydraulic integrators,^3,4^ fluid mappers,^5,6^ fluidic logic,^7,8^ reaction–diffusion computers,^9^ fluid maze solver,^6^ and liquid marbles logic.^10^

Despite their potentially nonlinear and computationally rich behavior, colloids have not been previously considered unconventional computing devices. Earlier, the concept of liquid cybernetic systems was developed,^11^ introducing colloidal autonomous soft holonomic processors with autolographic features.^12^ Recently, the computational capabilities of Fe_3_O_4_ ferrofluid were demonstrated, showcasing its ability to recognize digits using an 8 × 8 pixel-grid data set. This ferrofluid system is programmable and readable via electrical signaling.^13^ This advancement signals a departure from conventional notions, revealing the untapped potential of colloids in the realm of unconventional computing and offering new avenues for exploration and innovation.

In this work, we explore the capabilities of colloidal mixtures of zinc oxide (ZnO) and proteinoids. ZnO, a nontoxic biocompatible,^14^ being a direct band gap semiconductor with a large exciton binding energy of 60 meV,^15^ is considered a capable semiconductor to be used in numerous applications such as solar cells,^16−18^ photocatalysis,^19^ catalysts,^20^ and LEDs.^21^ Its nonsymmetric hexagonal wurtzite structure^22^ enables excellent piezoelectric,^23^ pyroelectric properties,^24^ and memristance.^25^ Our group has also conducted controlled experiments to reveal how ZnO colloids can act as electrical-analogue neurons, demonstrating synaptic-like learning^26,27^ and Pavlovian reflexes.^28^

Recent studies have demonstrated that when proteinoids are assembled in an aqueous solution, they can display intriguing computational capabilities.^29,30^ Exploring the mechanisms by which proteinoids can store and manipulate information may uncover novel biomolecular computing models that can be applied in bioinspired engineering.^31−34^ Colloidal proteinoids, similar to neural networks, demonstrate the ability to process inputs in a manner that resembles that of biological computing. Exploiting the computational capacity of colloidal systems through the development of customized proteinoids could pave the way for novel and unconventional computing devices.^35^ Additional examination of proteinoids will provide insights into prebiotic chemistry, biomolecular computing, and potentially a novel cohort of bioinspired computational systems.^36^

To formally assess the computational abilities of colloids beyond previous studies, we tested the implementation of Boolean logic in colloidal material. We applied binary strings by electrically stimulating the liquid, recorded the resulting electrical output responses, classified them, and finally obtained the logical expressions. This approach is inspired by *in-materia* computing^37,38^ techniques that characterize the properties of computational substrates. The exact approach, however, is based on simplified, bare-bone techniques of extracting Boolean functions, developed in computational experiments with acting computing devices^39^ and experimental laboratory implementation of fungal Boolean circuits.^38^

## Results and Discussion

### Fabrication and Characterization of Colloidal Solutions

Colloidal solutions of ZnO, proteinoids, and a mixture of proteinoids and ZnO were synthesized following the methodology detailed in Materials and Methods.

### Structural and Electronic Properties of the Colloidal Mixture

**Figure 1:**
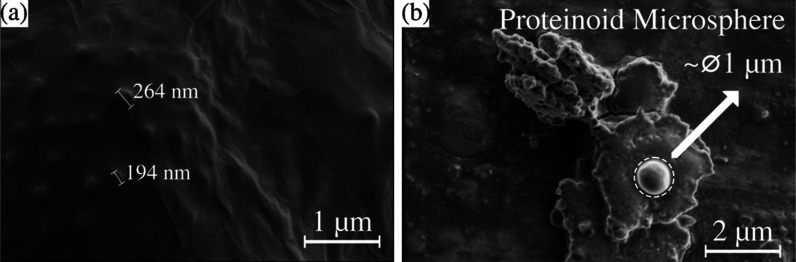
SEM images of (a) region rich in ZnO and (b) proteinoid microspheres in the mixture of ZnO and proteinoids.

The samples were drop-cast onto a Cu substrate of ∼200 μm at room temperature and left to dry for around 4 days to fabricate a thin layer of the mixture for scanning electron microscopy (SEM) imaging. SEM images reveal the agglomeration of ZnO nanoparticles during sample preparation (see Figure 1a). It is likely that surface tension causes the nanoparticles to realign as the solvent evaporates, resulting in most ZnO particles appearing layered. In the sample consisting of a mixture of ZnO and proteinoids, large proteinoid particles were observed to clump together with spots of agglomerated ZnO (Figure 1a) and proteinoid microspheres (Figure 1b). The morphology of the dispersed ZnO and proteinoid solutions revealed dispersed ZnO nanoparticles and proteinoid microspheres with diameters around 1 μm (see Figure 1b).

**Figure 2:**
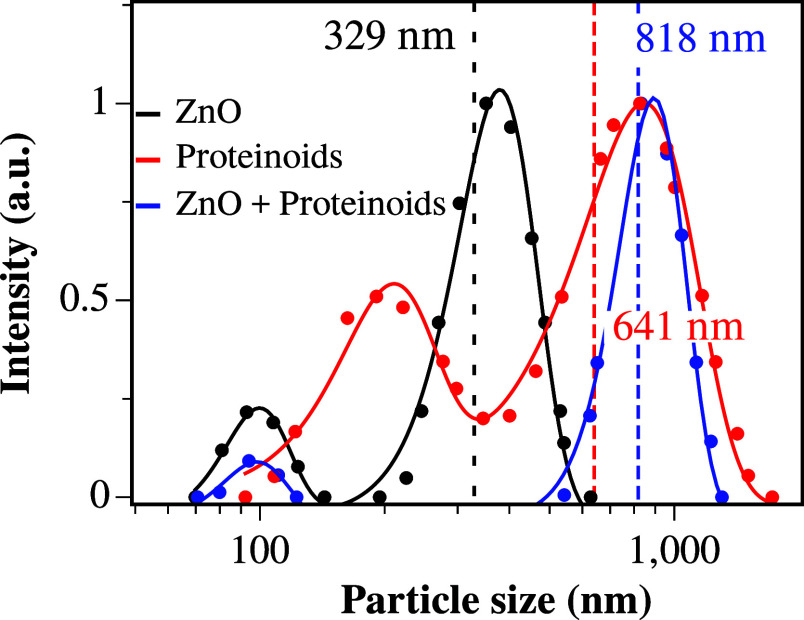
Particle size distribution and average particle size for ZnO colloidal suspension, dispersed proteinoids, and a mixture of both.

Dynamic light scattering analysis measured the particle size distribution of the colloidal suspensions (Figure 2). The average particle sizes for ZnO, proteinoids, and their mixture are 329, 641, and 818 nm, respectively. These results support the data in Figure 1b, indicating the agglomeration of ZnO nanoparticles on the proteinoid spheres, as evidenced by the increased particle diameter. The interaction between particles in the colloids likely arises from Brownian motion, facilitating electrical interactions between particles.^40,41^

**Figure 3:**
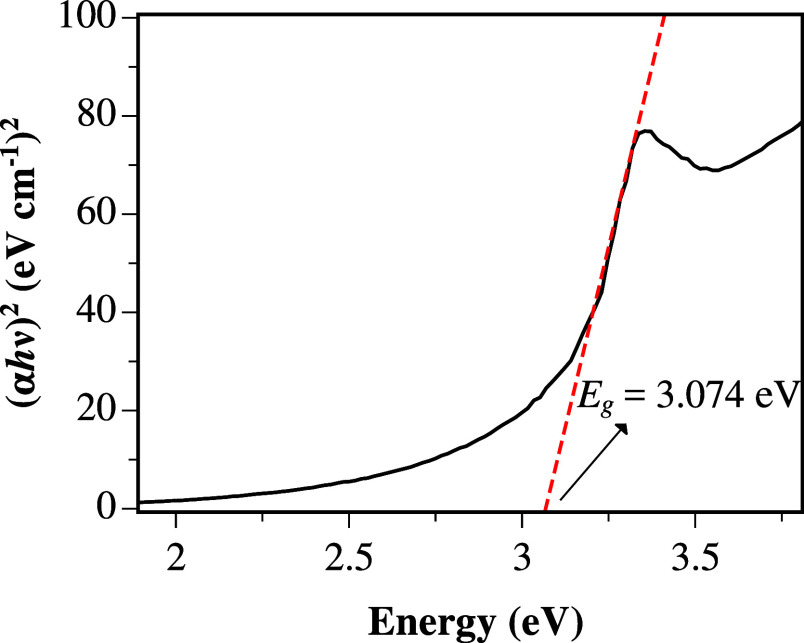
Tauc plot showing the reflectance spectrum versus photon energy for ZnO. The extrapolation of the optical band gap is represented by the dashed red line.

UV–vis spectroscopy was used to study the optical properties of the ZnO semiconductor. The Tauc method^42^ was employed to calculate the optical band gap, which is based on the following equation
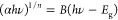
1where *h* is the Planck constant, ν is the photon frequency, *E*_g_ is the band gap, and *B* is a constant. The value of *n* is determined by the nature of the carrier transition, being equal to 1/2 or 2 for direct and indirect band gaps, respectively. Since ZnO is a direct band gap semiconductor,^43,44^*n* = 1/2 was used. The optical band gap is then estimated from a linear fit of (α*hν*)^1/*n*^ against the photon energy (*hν*) near the absorption edge. Figure 3 shows the reflectance spectrum of ZnO mapped according to eq 1 plotted against the photon energy. The linear fit of the data indicates a band gap of ∼3.074 eV, which is in agreement with the reported values of 3.1–3.37 eV.^45−47^

**Figure 4:**
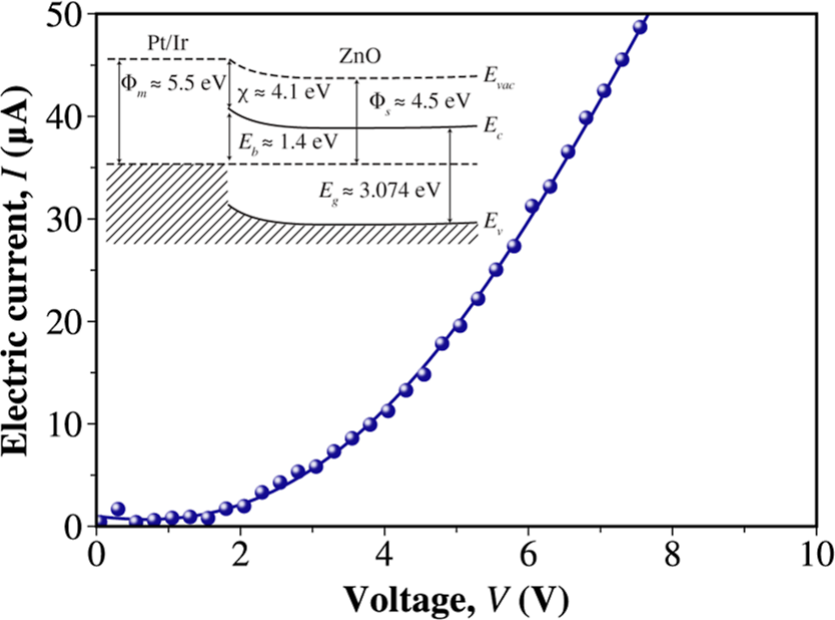
I quadrant portion of the *I*–*V* curve recorded from the colloidal solution. The inset represents the proposed band structure of the Pt/Ir contact with the ZnO nanoparticles.

The *I*–*V* characteristics of the colloidal suspension are shown in Figure 4. The exponential-like shape of the *I*–*V* curve demonstrates the occurrence of a Schottky barrier between the Pt/Ir probes and the ZnO nanoparticles.^48^ In the inset in Figure 4, the proposed band structure of the metal/semiconductor contact is drawn. The values for the work functions of the Pt/Ir alloy (Φ_m_ ≈ 5.5 eV^49^) and ZnO (Φ_s_ ≈ 4.5 eV^50^) and electron affinity of ZnO (χ ≈ 4.1 eV^51^) were obtained from the literature, while the band gap was experimentally measured as described above. The barrier height of *E*_b_ = 1.4 eV was estimated using the Schottky–Mott rule (*E*_b_ ≈ Φ_m_ – χ).^52^ This potential barrier^48^ between the Pt/Ir probes and the suspension indicates a strong nonlinear electrical behavior, and this nonlinearity is advantageous for implementing complex logic gates.^53,54^

### Logical Circuit Extraction

**Figure 5:**
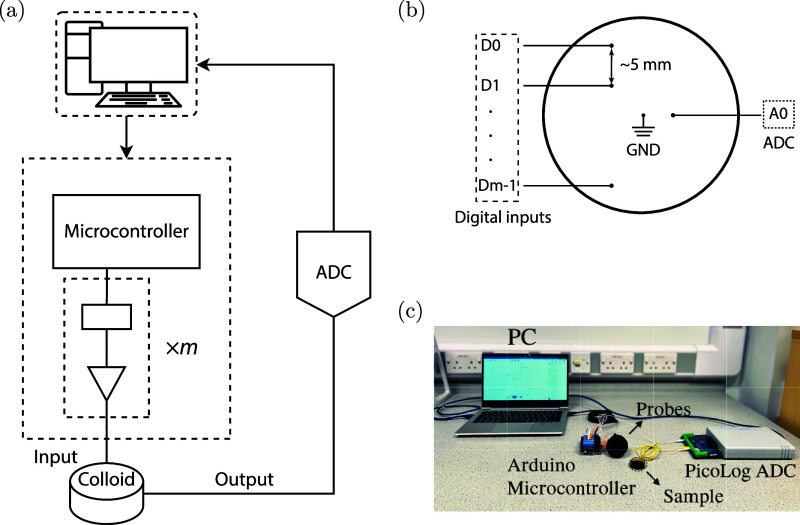
(a) Logic circuit extraction experimental scheme, where ADC refers to the analog-to-digital converter, (b) probe placement in the samples, and (c) picture of the experimental setup for 2-bit extraction.

Logical functions were extracted based on the theoretical methodology proposed in^31,39^ using an in-house device constructed around a microcontroller board (Arduino Uno, Arduino), as illustrated in Figure 5a. The strings were encoded in line with the unipolar return-to-zero logic, wherein a logical 0 (false) was encoded as 0 V, and a logical 1 (true) was encoded as 5 V. A series of 2, 4, and 8 Pt/Ir electrodes, each with a diameter of 10 μm, were positioned at a distance of approximately 5 mm from one another to extract logic gate circuits of 2-, 4-, and 8-bit, respectively (as seen in Figure 5b). Two additional Pt/Ir electrodes, also with a diameter of 10 μm and separated by approximately 5 mm, were placed in parallel to measure the output potential. The output electrodes were connected to a 24-bit analog-to-digital converter (ADC, ADC-24, PICO Technology). A picture showing the experimental setup for 2-bit logic function extraction is shown in Figure 5c.

A series of binary strings (*b* = {0,1}^*m*^, where *m* = 2, 4, and 8) were applied to the colloidal mixture. In all binary strings, each bit was changed every 15 s. The output voltage was sampled at a frequency of 17 Hz.

**Figure 6:**
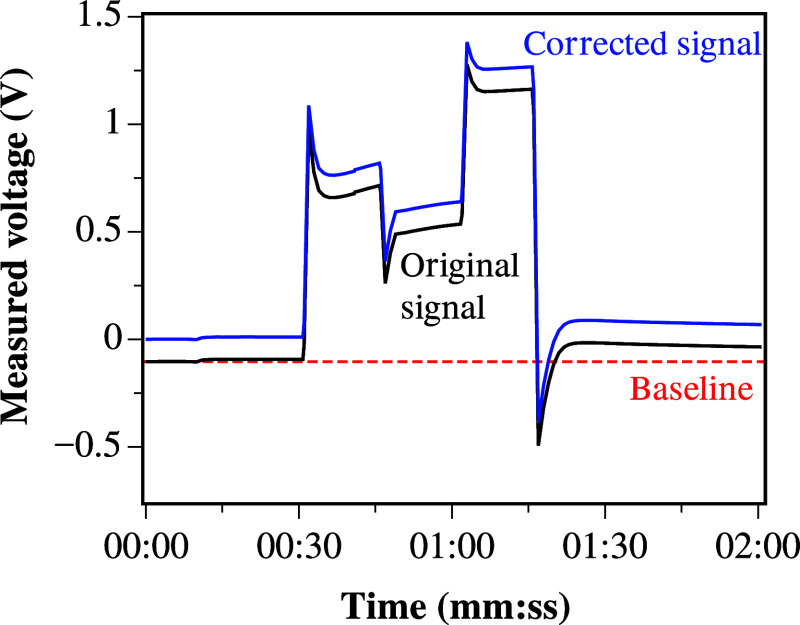
Measured output voltage and baseline correction of a ZnO and proteinoid mixture for a 2-bit string input.

The measured voltage output of the binary string *b* = {0,1}^2^ is depicted in Figure 6. The measurements showed a constant DC bias, as can be seen in Figure 6. The baseline was corrected by fitting a first-order polynomial using the procedure as described in.^55^

Logical circuits were extracted from the recorded data by classifying the measured electrical pulses according to a thresholding procedure. Each detected pulse was assigned a logical 1 (true) if its amplitude was greater than or equal to the selected threshold or a logical 0 (false) if it was below the threshold. Thresholds were selected from 5% of the peak voltage to 100% in 2.5% increments. The classified binary pulse sequences were stored in truth tables with each row representing a unique input–output combination observed in the experiment. The minimal sum-of-products (SOP) Boolean logic expression matching each truth table was then determined using the well-established Espresso logic minimization algorithm.^56,57^ From the computed minimal SOP expressions, the corresponding digital logic circuit was then realized.

**Figure 7:**
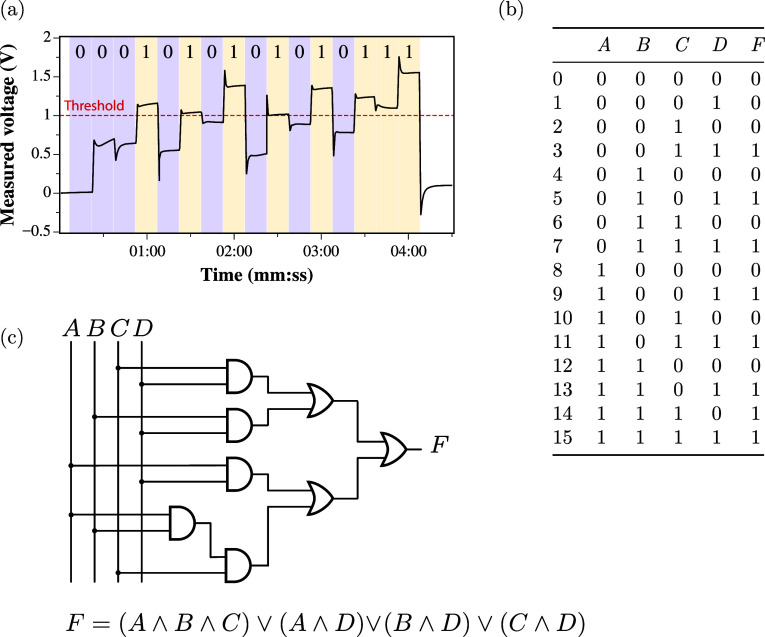
(a) Pulse classification for a 4-bit string, (b) resulting truth table for classified pulses, and (c) extracted SOP logic Boolean expression and realized logical circuit.

Figure 7 outlines the entire workflow from pulse measurement to logic circuit extraction. Figure 7a displays the classification procedure for 4-bit string input, Figure 7b shows the extracted truth table, and Figure 7c shows the realized logic circuit and fitted SOP.

### ZnO

Without repetition, a total of 2, 5, and 27 unique standard canonical SOP Boolean logical expressions were obtained for the experiments for the ZnO colloidal suspension with 2-, 4-, and 8-bit inputs, respectively. Tables 1–3 show the four most frequent logical expressions identified for each input bit width. As anticipated, the logical complexity of the most common expressions increased with the number of input variables. For 2-bit inputs, the most frequent expression was a simple disjunctive (OR) term. However, for 4-bit inputs, the most common expressions were composed of four disjunctive terms. The most frequent expressions for 8-bit inputs consisted of seven disjunctive terms and negated variables.

**Table 1:** Four Most Common Extracted Boolean Expressions for a 2-Bit String Input with Varying Thresholds for the ZnO Colloidal Suspension

SOP	count
*A* ∨ *B*	35
*A* ∧ *B*	3

**Table 2:** Four Most Common Extracted SOP Boolean Expressions for a 4-Bit String Input with Varying Thresholds for the ZnO Colloidal Suspension

SOP	count
*A* ∨ *B* ∨ *C* ∨ *D*	16
(*A* ∧ *B* ∧ *D* ∧ ¬ *C*) ∨ (*C* ∧ *D* ∧ ¬ *A* ∧ ¬ *B*)	6
(*C* ∧ *D*) ∨ (*A* ∧ *B* ∧ *D*)	4
*A* ∨ *B* ∨ *D*	2

**Table 3:** Four Most Common Extracted SOP Boolean Expressions for an 8-Bit String Input with Varying Thresholds for the ZnO Colloidal Suspension

SOP	count
¬ *A* ∨ ¬ *B* ∨ ¬ *C* ∨ ¬ *D* ∨ ¬ *E* ∨ ¬ *F* ∨ ¬ *G*	4
*A* ∧ *C* ∧ *D* ∧ *E* ∧ *F* ∧ *G* ∧ *H* ∧ ¬ *B*	2
*A* ∨ *C* ∨ *D* ∨ *E* ∨ *F* ∨ *H* ∨ (*B* ∧ ¬ *G*) ∨ (*G* ∧ ¬ *B*)	1
*C* ∨ *D* ∨ *E* ∨ *F* ∨ *H* ∨ (*A* ∧ *B*) ∨ (*A* ∧ ¬ *G*) ∨ (*B* ∧ ¬ *G*) ∨ (*G* ∧ ¬ *A* ∧ ¬ *B*)	1

**Figure 8:**
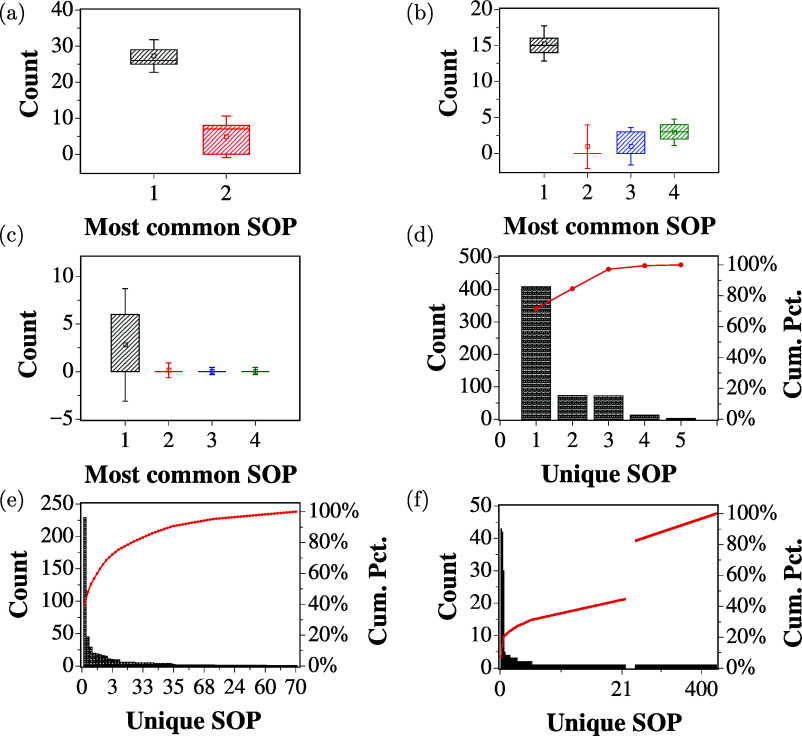
Boxplots showing the distribution of the four most common SOP expressions found in repeated experiments with (a) 2-bit, (b) 4-bit, and (c) 8-bit binary string input for the ZnO colloidal suspension. Whiskers indicate 1.5 times the standard deviation. Pareto charts showing the frequency counts of unique SOPs from the repeated experiments for (d) 2-bit, (e) 4-bit, and (f) 8-bit binary string input. The red curve represents the cumulative percentage of the total count.

The reproducibility of the extracted logic circuits was evaluated by conducting 15 repetitions of the experiments for each binary input string. Before each repetition, the sample was manually shaken to disrupt any existing structures. Figure 8a–c displays boxplots summarizing the results for the most common SOPs seen in Tables 1–3 for 2-, 4-, and 8-bit, respectively, with the whiskers illustrating 1.5 times the standard deviation across the repeats. The repeatability of the most common logic expressions across the repeats establishes the ability of the colloidal solution to produce circuits that are reproducible for a given input sequence.

To analyze the distribution of unique SOPs during the repeats, Pareto plots representing the results of the multiple trials conducted for the 2-, 4-, and 8-bit experiments were plotted and are shown in Figure 8d–f. These plots are a type of histogram that arranges the results by frequency to visualize their distribution and relative dominance. The occurrence of a large number of SOPs with high frequency demonstrates that the number of different SOP expressions increases as the number of input bits increases, reflecting the greater number of possible logic functions that can be realized.

The plots exhibit a strong positive skewness, indicating that a large set of thresholds produced repeated results for all trials with the same set of most common logic functions (as seen in Tables 1–3) This highlights the robust nature of the obtained logic functions. The binary thresholding classification procedure used in this work^58^ means that as the number of bits increases, the probability of incorrect classification also increases due to noise in the signal.^59,60^ This is supported by the increased occurrence of unique SOPs with lower numbers in the 4- and 8-bit experiments. However, the results demonstrate the repeatability of the findings, as evidenced by the dominance of a very small set of unique SOPs (as shown by the red curves in Figure 8d–f).

### Proteinoids

The experiments were repeated for 2-, 4-, and 8-bit inputs using the proteinoid suspension, and a total of 3, 9, and 17 unique SOP expressions were found, respectively. Tables 4–6 show the four most common logical expressions identified for each input binary string. The logical complexity of the most common expressions increased with more input variables, although at rates different from those in the previous ZnO suspension experiments. For 2-bit inputs, the most common expression was a logical 0 term, whereas in the ZnO case, it was a disjunctive term, showing a clear contrast between the materials. The most common expressions for 4-bit inputs showed a lower circuit complexity (lower number of extracted logic gates) when compared to those shown for the ZnO colloidal suspension. The most common expressions for 8-bit showed a large number of disjunctive terms, indicating a higher level of circuit complexity than that of the ZnO suspension. While the complexity increased as more inputs were added, both the expressions and their frequency highlighted the differences between the materials.

**Table 4:** Four Most Common Extracted SOP Boolean Expressions for a 2-Bit String Input with Varying Thresholds for the Dispersed Proteinoids

SOP	count
0 (false)	19
*A* ∨ *B*	16
*A* ∧ *B*	3

**Table 5:** Four Most Common Extracted SOP Boolean Expressions for a 4-Bit String Input with Varying Thresholds for the Dispersed Proteinoids

SOP	count
*A* ∨ *B* ∨ *C* ∨ *D*	23
*A* ∧ *B* ∧ *C* ∧ *D*	4
*A* ∨ *B* ∨(*C* ∧ *D*)	3
(*A* ∧ *B*) ∨ (*B* ∧ *D*) ∨ (*C* ∧ *D*) ∨ (*A* ∧ ¬ *C* ∧ ¬ *D*)	3

**Table 6:** Four Most Common Extracted SOP Boolean Expressions for an 8-Bit String Input with Varying Thresholds for the Dispersed Proteinoids

SOP	count
¬ *A* ∨ ¬ *B* ∨ ¬ *C* ∨ ¬ *D* ∨ ¬ *E* ∨ ¬ *F* ∨ ¬ *G* ∨ ¬ *H*	19
(*A* ∧ ¬ *E*) ∨ (*B* ∧ ¬ *H*) ∨ (*C* ∧ ¬ *G*) ∨ (*D* ∧ ¬ *F*) ∨ (*E* ∧ ¬ *D*) ∨ (*F* ∧ ¬ *C*) ∨ (*G* ∧ ¬ *B*) ∨ (*H* ∧ ¬ *A*)	4
*A* ∧ *B* ∧ *C* ∧ *D* ∧ *E* ∧ *F* ∧ *H* ∧ ¬ *G*	2
(*C* ∧ ¬ *B*) ∨ (*C* ∧ ¬ *D*) ∨ (*D* ∧ ¬ *E*) ∨ (*E* ∧ ¬ *G*) ∨ (*F* ∧ ¬ *H*) ∨ (*G* ∧ ¬ *F*) ∨ (*H* ∧ ¬ *E*) ∨ (*A* ∧ *B* ∧ ¬ *C*) ∨ (*A* ∧ *H* ∧ ¬ *B*) ∨ (*B* ∧ *H* ∧ ¬ *C*)	1

**Figure 9:**
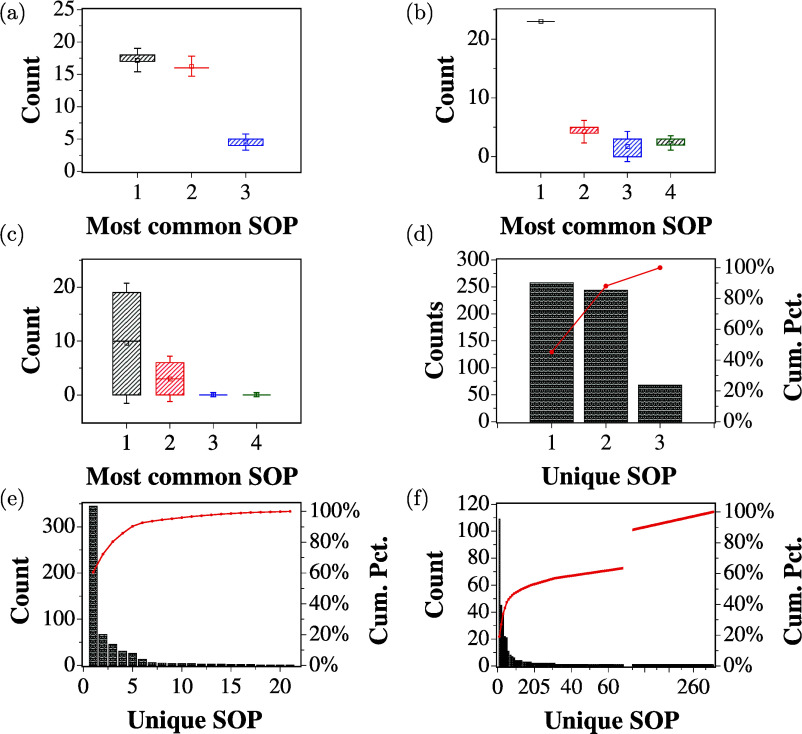
Boxplots showing the distribution of the four most common SOP expressions found in repeated experiments with (a) 2-bit, (b) 4-bit, and (c) 8-bit binary strings for the dispersed proteinoid. Whiskers indicate 1.5 times the standard deviation. Pareto charts showing the frequency of unique SOPs from the repeated experiments for (d) 2-bit, (e) 4-bit, and (f) 8-bit binary string input. The red curve represents the cumulative percentage of the total count.

The reproducibility of the extracted logic circuits using proteinoids was assessed across 15 repetitions of experiments for each binary input string. This was conducted similarly to the prior ZnO colloidal suspension experiments, wherein the samples were shaken before each repetition to disrupt the existing structures. Boxplots in Figure 9a–c illustrate the outcomes for 2-, 4-, and 8-bit inputs, with whiskers denoting 1.5 times the standard deviation across the repeats. Similarly to what was seen in the previous experiments involving the ZnO colloidal suspension, the consistent occurrence of the most frequent logical expressions during the repetitions demonstrates the ability to create logical circuits in a deterministic manner.

The Pareto charts in Figure 9d,e illustrate the results obtained from replicates by enumerating the frequency of occurrence of unique logic functions across all repetitions of 2-, 4-, and 8-bit experiments. The charts demonstrate the repeatability of the results with a limited set of unique logic functions dominating across all repetitions, similar to the observations in the ZnO colloidal suspension experiments.

### Mixture of ZnO and Proteinoids

Having studied the properties of both the ZnO colloidal suspension and dispersed proteinoids, we now evaluated the properties of their mixture. Following the same procedure as before, a total of 4, 6, and 24 unique standard canonical SOP Boolean logical expressions were obtained for the 2-, 4-, and 8-bit input experiments. The four most frequent logical expressions identified for each input binary string are displayed in Tables 7–9. Again, the logical complexity of the frequent expressions escalated with greater input variables, however, in a manner distinct from that of either the ZnO colloidal suspension or the dispersed proteinoids.

**Table 7:** Four Most Common Extracted SOP Boolean Expressions for a 2-Bit String Input with Varying Thresholds for the Mixture of ZnO and Proteinoids

SOP	count
*A* ∧ *B*	21
*A* ∨ *B*	14
*B*	2
*A*	1

**Table 8:** Four Most Common Extracted SOP Boolean Expressions for a 4-Bit String Input with Varying Thresholds for the Mixture of ZnO and Proteinoids

SOP	count
¬ *A* ∨ ¬ *B* ∨ ¬ *C*	15
(*A* ∧ ¬ *B*) ∨ (*B* ∧ ¬ *C*) ∨ (*D* ∧ ¬ *A*)	7
*A* ∧ *B* ∧ *D* ∧ ¬ *C*	5
(*A* ∧ *B* ∧ ¬ *C*) ∨ (*A* ∧ *D* ∧ ¬ *B*) ∨ (*B* ∧ *D* ∧ ¬ *C*)	4

**Table 9:** Four Most Common Extracted SOP Boolean Expressions for an 8-Bit String Input with Varying Thresholds for the Mixture of ZnO and Proteinoids

SOP	count
*A* ∨ *B* ∨ *C* ∨ *D* ∨ *E* ∨ *F* ∨ *G* ∨ *H*	6
*A* ∧ *B* ∧ *C* ∧ *D* ∧ *E* ∧ *F* ∧ *G* ∧ ¬ *H*	3
(*A* ∧ ¬ *D*) ∨ (*B* ∧ ¬ *G*) ∨ (*C* ∧ ¬ *F*) ∨ (*D* ∧ ¬ *E*) ∨ (*E* ∧ ¬ *C*) ∨ (*F* ∧ ¬ *B*) ∨ (*G* ∧ ¬ *A*) ∨ (*G* ∧ ¬ *H*)	2
(*A* ∨ *C* ∨ *D* ∨ *E*) ∨ (*B* ∧ *F*) ∨ (*B* ∧ *G*) ∨ (*B* ∧ *H*) ∨ (*F* ∧ *G*) ∨ (*F* ∧ *H*) ∨ (*G* ∧ *H*)	1

For 2-bit inputs, the dominant expression was a simple conjunctive term, in contrast to the disjunctive forms seen previously for ZnO suspension and logical 0 for dispersed proteinoids. The most common 4-bit input expressions comprised three disjunctive and negated clauses, while the sample with just ZnO yielded four disjunctive and eight conjunctive terms. The most frequent 8-bit input expressions consisted of eight disjunctive and negated variables, exhibiting intermediate complexity. The unique nature of the extracted logic functions and their corresponding frequencies for each material suggest that each material has a distinct response.

**Figure 10:**
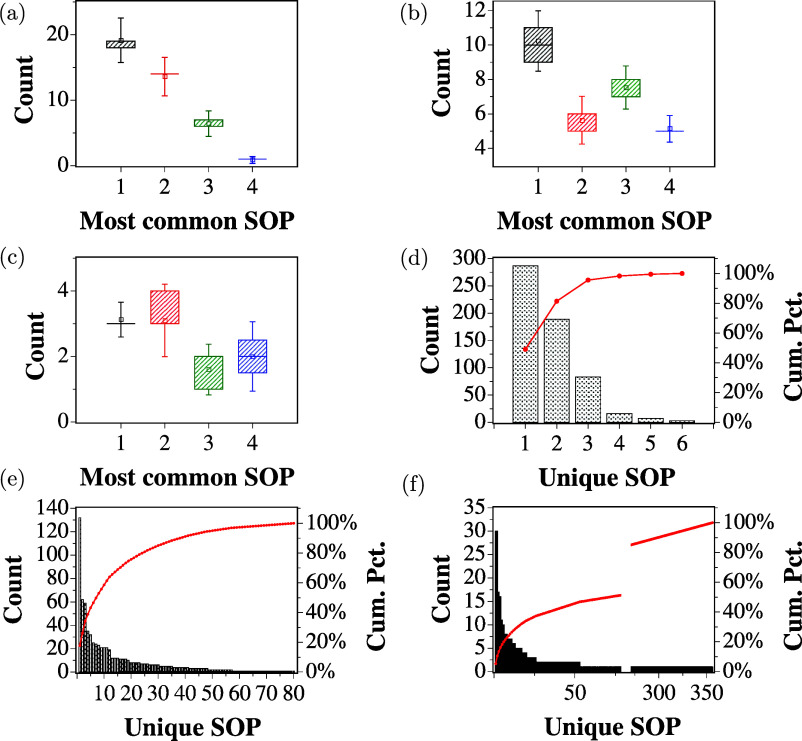
Boxplots showing the distribution of the four most common SOP expressions found in repeated experiments with (a) 2-bit, (b) 4-bit, and (c) 8-bit binary string input for the mixture of ZnO and proteinoids. Whiskers indicate 1.5 times the standard deviation. Pareto charts showing the frequency of unique SOPs from the repeated experiments of (d) 2-bit, (e) 4-bit, and (f) 8-bit binary string input. The red curve represents the cumulative percentage of the total count.

The reproducibility of the extracted logic circuits using the ZnO and proteinoid mixture was tested over 15 repetitions for each binary input string, with shaking before each repetition, following the same methodology as before. Boxplots for 2-, 4-, and 8-bit inputs are shown in Figure 10a–c, with whiskers indicating 1.5 times the standard deviation across repetitions. The recurrence of common logical expressions during the repeats emphasizes that the circuits are deterministically derived from the mixture for a given binary string, similar to what was seen for the individual components of the mixture.

The Pareto charts in Figure 10d–f demonstrate the reproducibility of the results, as a limited set of distinctive logic functions consistently dominate across all repeats, similarly to what was observed for the individual components.

### Complexity Comparison

To investigate the impact of the mixture on the complexity of extracted logic functions, we determined the number of clauses in the conjunctive normal form representation for all unique SOPs obtained across 15 repetitions of each experiment for the ZnO colloidal suspension, dispersed proteinoids, and their mixture. The results are presented in Figure 1. Although it was expected that there would be a large increase in complexity due to potential nontrivial interactions between the two materials, the observed complexity in the mixed material appears to be a weighted combination of the complexities of the individual components.

**Figure 11:**
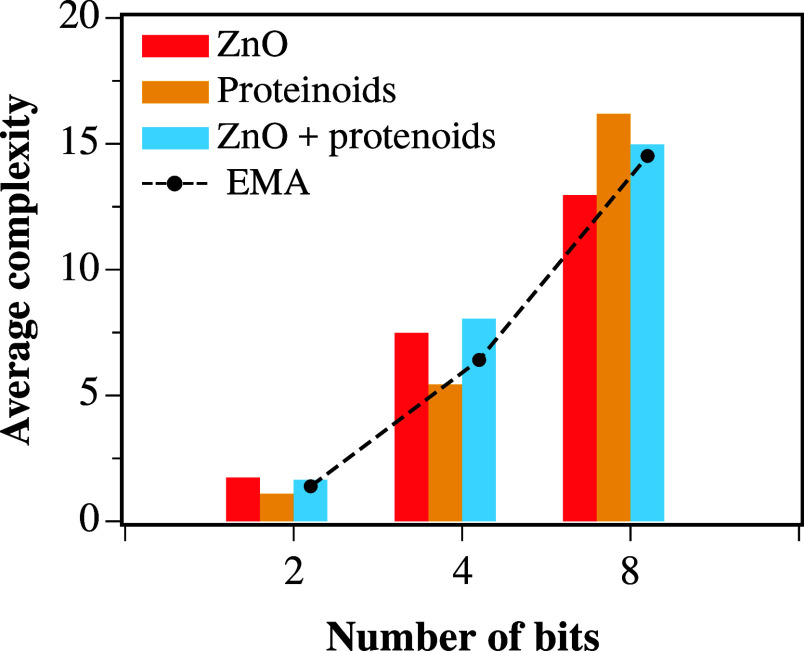
Complexity of the obtained logical expressions for ZnO colloidal suspension, dispersed proteinoids, and the mixture of both for 2-, 4-, and 8-bit binary input strings.

A straightforward approach to exploring this is to use the effective medium approximation. This method approximates the bulk properties of a composite material by using a quadratic equation that weighs the properties of the mixture based on the properties and fraction of each component. This approach has been previously used to provide reasonable estimates for the electrical resistivity and thermal conductivity of multiphase materials.^61,62^ For a binary system, the effective medium approximation is given as^63^
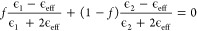
2where *f* is the fraction of the first component of the mixture, ϵ_1_ is the property of the first component of the mixture, ϵ_2_ is the property of the second component of the mixture, and ϵ_eff_ is the effective component of the mixture. The predicted complexity values from this simple model are shown by the black circles in Figure 11.

The estimated complexity is in good agreement with the measured values, except for the estimated complexity of the logic functions extracted using a 4-bit binary string input, where the calculated value is less than the measured one. Although the values are not an exact match, likely due to the simplicity of the model, the trend is observable and suggests that a reasonable estimate of logic complexity can be obtained using this setup by considering the individual complexity of each component.

## Conclusions

In this work, the capabilities of colloidal suspensions of ZnO, proteinoids, and their mixtures were investigated using a bare-bones experimental setup. We demonstrate the feasibility of studying and using these materials for computational purposes. In particular, we verify unique responses and complexities for each material with the combined system exhibiting averaged rather than enhanced complexity. While ZnO yields more complex logic expressions, in general, proteinoids enable a more robust response. This research highlights colloidal suspensions as promising materials for unconventional computing while illustrating the nontrivial relationship between device complexity and material behavior. Further study may reveal ways to tune and optimize the computational repertoires of multicomponent colloidal logic.

## Materials and Methods

### Preparation of ZnO Suspension

Sodium dodecyl sulfate (SDS, Merck) was added to deionized water and stirred to create a homogeneous surfactant solution with a concentration of 0.22 wt %. Under stirring, 2 mL of the solution and 1 mL of NaOH (Reagent grade, Merck, ≥98%) were added to 7 mL of dimethyl sulfoxide (DMSO, Pharmaceutical grade 99.9%, Fisher Scientific). Then, 3 mg of ZnO nanoparticles (+99%, 10–25 nm, PlasmaChem) was added to the mixture while constant stirring, with a resulting dispersion concentration of 0.3 mg mL^–1^. The resulting suspension was placed in an ultrasonic bath (DK Sonic Ultrasonic, 40 kHz) for 30 min. After ultrasonication, stirring was repeated for a few hours to ensure a homogeneous dispersion of ZnO.

### Preparation of Proteinoids

Proteinoids were synthesized by mixing glycine (99.5%, Sigma-Aldrich), alanine (99.5%, Sigma-Aldrich), aspartate (99.5%, Sigma-Aldrich), and glutamate (99.5%, Sigma-Aldrich) in a reaction flask. This combination was thermally polymerized at 433 to 453 K, where these elevated temperatures activated condensation processes between amino acid amine and carboxyl moieties. These condensation processes, which form peptide bonds, polymerize polypeptide chains of different lengths into proteinoids. The polymerization was stopped by cooling the reaction vessel to room temperature. Proteinoids precipitated from the solution after the cooled reaction mixture was dissolved in a 353 K aqueous medium. Lyophilization freeze-dries the precipitate to isolate the proteinoids from the aqueous phase. Before lyophilization, the solid was washed to remove impurities and recover the proteinoids. This multistage thermochemical process produces proteinoids, short protein-like polymer chains, from amino acid precursor monomers.

### Preparation of Mixture

The mixture of ZnO and proteinoids was created by combining equal volumes of each component. This was done to ensure a balanced and homogeneous composition and to equally evaluate the unique properties of ZnO and proteinoids.^30^

### Suspension Characterization

The morphology of the suspensions was determined on an FEI Quanta 650 scanning electron microscope at 2 kV. Measurements of particle size and size distribution were performed with a Malvern Instruments Zetasizer Nano ZS at 25 °C. The absorbance of the samples at room temperature was measured using a PerkinElmer Lambda XLS ultraviolet–visible spectrometer. *I*–*V* characteristics of the samples were analyzed using a Keithley 2400 Source Measure Unit. All electrical measurements were conducted using Pt/Ir electrodes with a 10 μm of diameter.

## Data Availability

Data sets that support the findings of this study are available in the Zenodo database (10.5281/zenodo.12180078).
